# A 46-week outbreak of ertapenem-resistant, non-carbapenemase encoding *Klebsiella pneumoniae* ST45 in a paediatric cardiac unit involving shared equipment, United Kingdom, April 2022 to February 2023

**DOI:** 10.2807/1560-7917.ES.2025.30.43.2500133

**Published:** 2025-10-30

**Authors:** Alice J Fraser, Christopher M Parry, Beatriz Larru, Lindsay Case, Kate Ball, Caitlin Duggan, Thomas Edwards, Eva Heinz

**Affiliations:** 1 Department of Tropical Disease Biology, Liverpool School of Tropical Medicine, Liverpool, United Kingdom; 2 Department of Microbiology, Alder Hey Children’s NHS Foundation Trust, Liverpool, United Kingdom; 3 Department of Clinical Sciences, Liverpool School of Tropical Medicine, Liverpool, United Kingdom; 4 Department of Infection Prevention and Control, Alder Hey Children’s NHS Foundation Trust, Liverpool, United Kingdom; 5 Department of Vector Biology, Liverpool School of Tropical Medicine, Liverpool, United Kingdom; 6 Strathclyde Institute of Pharmacy and Biomedical Sciences, University of Strathclyde, Glasgow, United Kingdom

**Keywords:** infection control, nosocomial outbreak, hospital equipment, epidemiology, beta-lactamase

## Abstract

An outbreak of an ertapenem-resistant *Klebsiella pneumoniae* clone occurred in a specialist children’s hospital in Liverpool, United Kingdom (UK), from April 2022 to February 2023. Carbapenem-resistant *K. pneumoniae* is unusual in the UK, and identification of two isolates exhibiting ertapenem resistance in the same ward in December 2022 raised concerns and triggered an outbreak investigation. Potential transmission through shared equipment was identified; a total of 11 patients were colonised and/or infected by phenotypically similar isolates. Multilocus sequence typing supported hospital transmission, and short-read whole genome sequencing (WGS) was performed on all isolates; long-read sequencing was conducted for three isolates to confidently resolve the plasmid structure. WGS confirmed a clonal outbreak and strongly supported the suspected nosocomial transmission. Detailed analysis of the resistance determinants indicated that ertapenem resistance was driven by a combination of different beta-lactamases, which would not alone convey this resistance profile, along with modifications in porin structure that suggested a synergistic interaction. These findings highlight how highly resistant strains could be mislabelled as predicted sensitive when considering genetic determinants in isolation and underscore the need to study beta-lactam resistances beyond the presence or absence of specific genes but also to consider co-occurrence.

**Key public health message**

**What did you want to address in this study and why?**

*Klebsiella pneumoniae* (*Kpn*) bacteria can cause serious infections and are often transmitted in hospitals. We investigated an outbreak of infections caused by *Kpn* resistant to multiple antibiotics in a children’s hospital in Liverpool, United Kingdom, occurring from April 2022 to February 2023. To understand potential sources of infection, we examined the wards, patient locations and equipment use.

**What have we learnt from this study?**

The genome sequence analysis of bacteria isolated from patient samples confirmed that the *Kpn* infections were most likely the result of within-hospital transmission events. We identified healthcare equipment shared between the group of involved patients, which likely served as reservoir.

**What are the implications of your findings for public health?**

Identification of shared equipment as a possible transmission route re-emphasises the role of the infection prevention and control team in outbreak management. Findings further highlight the importance of genome sequencing to define *Kpn* similarity between infected patients and awareness that new resistance gene combinations could reflect unexpected bacterial antimicrobial resistance profiles.

## Background

*Klebsiella pneumoniae* (*Kpn*) is a major cause of healthcare-associated infections globally. While *Kpn* can be found in healthy individuals as part of the gut microbiome, it can cause a spectrum of illnesses including urinary tract infections, pneumonia and bacteraemia [1]. An increasing number of *Kpn* strains causing healthcare-associated infections are resistant to multiple classes of antimicrobials, including the commonly prescribed beta-lactams [2,3]. This growing resistance complicates patient management, leading to delays in effective treatment and often necessitating the use of last-line antimicrobials which further contributes to poor clinical outcomes [4,5] and increased healthcare costs [6].

Hospitals serve as a conduit for the evolution and transmission of *Kpn*, particularly to susceptible patient populations with compromised immune systems and complex medical needs [7,8]. Hospital environments provide unique selection pressures, promoting the survival and proliferation of resistant strains as a result of the extensive use of antibiotics and the proximity of colonised or infected individuals to other vulnerable patients [9]. The expansion of specific *Kpn* lineages that have accumulated multiple antimicrobial resistance genes (ARGs), such as those encoding extended-spectrum beta-lactamases (ESBL) from the SHV, TEM and CTX-M families, as well as AmpC enzymes and carbapenemases, is commonly associated with hospitals and healthcare settings [10]. Additionally, non-carbapenemase-producing strains may carry multiple beta-lactamases whose synergistic effects can produce unusual phenotypes, i.e. carbapenem resistance, especially when occurring in conjunction with chromosomal mutations, such as those affecting porins [11]. This resistance, in the absence of typical carbapenemase genes, may not be detected by molecular diagnostics.

The spread of these ARGs is facilitated by their frequent location on mobile genetic elements (MGEs), which enables horizontal transfer. This leads to enhanced resistance as ARGs accumulate and combine with other advantageous chromosomal mutations, promoting the rapid dissemination of multidrug resistance elements among *Kpn* [10,12]*.* These dynamics pose a notable challenge to both the diagnosis of infections and subsequent treatment, complicating clinical management and contributing to the ongoing threat of antimicrobial resistance.

### Outbreak detection

In December 2022, two infants in the cardiac ward of a children’s hospital in Liverpool, United Kingdom (UK) developed a bloodstream infection with an ertapenem-resistant strain of *Kpn.* Further investigation revealed that between April and December 2022, a total of 11 infants and children who were inpatients in the cardiac and the critical care wards were colonised, infected or both colonised and infected, with an apparently similar ertapenem-resistant strain of *Kpn.* A shared piece of equipment was identified as the most likely source of transmission, and cleaning efforts halted further spread. The last positive colonisation sample was taken at 56 weeks after admission of the first patient involved in the outbreak, at which point the outbreak was declared controlled. The isolates were retrospectively analysed using whole genome sequencing (WGS), confirming a clonal outbreak of *Kpn* sequence type (ST) 45.

Here, we present an overview of the outbreak and a detailed genomic investigation of the antimicrobial-resistant *Kpn.* Using WGS and comprehensive bioinformatics analyses, we explain the genetic underpinnings of resistance and highlight the intra-strain evolution that occurred within outbreak strains.

## Methods

### Outbreak setting

Hospital A is a specialist healthcare provider for over 330,000 children and young people each year. It is a regional centre for cardiology and cardiac surgery for the north-west of England and north Wales. Patients are cared for in the cardiac ward or the critical care ward according to clinical needs. The cardiac ward consists of 23 beds with a mixture of single-patient rooms with en-suite bathroom and four-bed bays with shared bathrooms. The critical care ward consists of bays with six beds widely spaced from each other. Each bay has a sluice. There are no designated hospital areas where relatives can meet outside of the wards. 

The patients in the wards are predominantly acutely unwell and include those pre- and post-operative. While not a long-term care facility, many of the children are in critical condition, and it is not unusual for them to be in the wards for long periods. The majority of patients are not severely immunocompromised but are considered a vulnerable group, and most have complex underlying cardiac abnormalities, some with other congenital abnormalities and some are neonates. Most patients had indwelling vascular catheters during their hospitalisation.

### Identification of outbreak cases and definitions

Routine screening of faecal samples or swabs for multidrug-resistant organisms (MDRO), Gram-negative bacteria with resistance to extended-spectrum cephalosporins, carbapenems and ciprofloxacin, are conducted on admission to the critical care ward and weekly during the in-patient stay. Blood cultures are taken as required in patients with fever or suspected sepsis. Samples were processed using standard microbiological methods and isolates identified using MALDI-TOF MS Biotyper (Bruker). 

A retrospective review of faecal surveillance results over the previous 12 months was performed by searching in the laboratory electronic information system for ertapenem- or meropenem-resistant *Kpn* isolates from any sample site among patients from the cardiac and critical care wards. Patients considered part of the outbreak were those colonised and/or infected with an ertapenem- or meropenem-resistant *Kpn* while in the cardiac or critical care ward between January 2022 and February 2023. Colonised patients were defined by the isolation of the *Kpn* from faecal MDRO surveillance cultures. Infected patients were defined as patients with the *Kpn* isolated from a blood culture, or culture of another sterile site. The organism was identified by MALDI-ToF and antimicrobial susceptibility tests. 

### Antimicrobial susceptibility testing

Initial antimicrobial susceptibility testing (AST) was performed using the disc diffusion (no broth enrichment) method according to EUCAST guidelines [13] for all antibiotics except ertapenem and meropenem, where susceptibility was determined using E-tests (bioMérieux, France) according to the manufacturer’s instructions and EUCAST breakpoints. Susceptibility testing of cefiderocol was repeated, using the broth microdilution method, according to EUCAST guidelines and using cation-adjusted Mueller Hinton broth (CA-MHB) made according to Hackel et al [14].

### Bacterial isolates

Clinical isolates were stored at −80 °C at the time of initial isolation and later transferred from the hospital to the Liverpool School of Tropical Medicine on agar slants under a Material Transfer Agreement. Isolates were sub-cultured on Luria-Bertani (LB) agar and then stored in glycerol broth in Microbank tubes (Pro-laboratory Diagnostics) at −80 °C. For subsequent experiments, isolates were recovered and plated on LB agar (Sigma) at 37 °C for 18 h, from which single colonies were then picked and used for subsequent experiments.

### Outbreak management

Outbreak management at the hospital included enhanced surveillance and contact tracing, prompt isolation of suspected and confirmed cases, re-enforcement of transmission-based precautions, education of staff, communication and enhanced environmental cleaning. The hospital does not have an environmental testing program.

### DNA extraction for whole genome sequencing

Single colonies were transferred to 10 ml of LB broth (Sigma), and incubated overnight at 37 °C, 200 RPM. Long fragments of genomic DNA, used for Oxford Nanopore Technologies (ONT) sequencing, were extracted using the MasterPure Complete DNA and RNA Purification Kit (Lucigen), following the manufacturer’s instructions for the purification of DNA from cell samples. DNA used for Illumina sequencing and qPCR was extracted using the DNeasy Blood and Tissue Kit (Qiagen), following the protocol for Gram-negative bacteria. The quality and size of the long-fragment DNA used for Illumina sequencing was assessed using the TapeStation (4150) system and the Genomic DNA ScreenTape Kit (Agilent). All genomic DNA used for sequencing was quantified using the Qubit Fluorometer with the dsDNA BR Kit and dsDNA HS Kit (Invitrogen).

### Whole genome sequencing

Short-read sequencing was performed by Microbes NG according to their methodology (https://microbesng.com/documents/methods). Long-read sequencing was performed on a MinION MK1B sequencing device (ONT). Library preparation was carried out according to the manufacturers protocol, using the ligation sequencing kit (SQK-LSK109) and Native Barcoding Expansion Kits (EXP-NBD104; all ONT). Sequencing was carried out using a FLOW-MIN106 R9.4.1 flow cell (ONT), the libraries were loaded as a pool containing a maximum of 10 sample libraries in total. Illumina and ONT reads can be found at the Sequence Read Archive bioproject ID PRJNA1211177. A detailed list of accession numbers is available in Supplementary Table S1.

### Assemblies

Short reads were adapter trimmed using Trimmomatic version 0.30 [15] with a sliding window quality cutoff of Q15 and assembled using Shovil (v1.1.0) [16]. Basecalling and de-multiplexing of long reads was performed with Guppy (v6.4.6) [17] using the super-accurate model for basecalling. Long read sequences were then filtered using Filtlong (v0.2.1) [18] and adapters trimmed using Porechop (v0.2.4) [19]. Long-read-first hybrid assemblies were produced using Flye (v2.9.3) [20], these were then visualised using Bandage (v0.8.1) [21], before being polished with long reads using Medaka (v1.8.0) [22]. Assemblies then underwent polishing with short reads using Polypolish (v0.6.0) [23] and Pypolca (v0.3.0) [24]. Finally, polished assemblies were quality checked, using BWA (v0.7.17) and the bwa-mem algorithm [25] and samtools (v1.9) [26] to map reads back to the consensus assembly sequence. These were then visualised using Artemis (v18.2.0) [27].

### Genome analyses

Polished assemblies were annotated using PROKKA (v.1.14.6) [22], any unannotated genes of interest were manually queried using BLAST. Sequence type, K type, O antigen group and virulence loci using Kleborate (v2.4.1) [28], with the Achtman [23] scheme for multilocus sequence typing (MLST) used. Plasmid replicons were determined using Plasmidfinder (v2.0.1) [26]. ARGs were identified in short reads using ARIBA (v2.14.6) [29], and the SRST2 ARGANNOT database. ABRICATE(v1.0.0) and the CARD [30] database was used to identify ARGs in long read-first hybrid assemblies. Chromosomal mutations were identified using ResFinder (v4.1). Plasmids were visualised using Proksee [31] and compared using the inbuilt BLAST [32] tool (v1.3.1). Core genome SNPs for isolates were determined using snippy (v4.6.0) [33], using isolate MB1 as the reference. Recombinant regions were then removed from the genome alignments using Gubbins (v2.3.4) [34], polymorphic sites were removed using SNP-sites (v.2.5.1) [35] and a Newick phylogenetic tree was produced using FastTree [36] and the General Time Reversible Model. The resulting tree was adjusted to SNP-length branch lengths using the Python implementation of pyjar (https://github.com/simonrharris/pyjar) [37].This was then imported using Phytools [38] and further analysed using rPinecone [39] which uses a root-to-tip approach to confidently distinguish closely related isolates based on their evolutionary lineage; new major lineages were defined by the presence of 10 single nucleotide variants (SNVs), and new minor lineages were determined by the presence of 5 SNVs which is a currently used community standard, although the best practice for defining a cutoff for transmission is still being studied and suspected transmission events should always be supported by clinical or epidemiological insights. The resulting trees were visualised in iTOL [40].

## Results

### Epidemiological investigation

**Figure 1:**
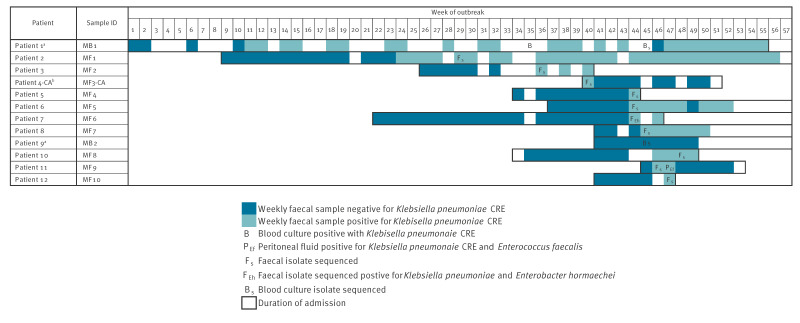
Timeline of hospital admission and detection in patients identified as colonised and/or infected with the outbreak strain of *Klebsiella pneumoniae*, United Kingdom, February 2022–February 2023 (n = 12)

CA: community-acquired; CRE: carbapenem-resistant Enterobacterales.

^a^ Patients 1 and 9 were initially identified.

^b^ The isolate from Patient 4 was positive for ertapenem-resistant *Kpn* at the time of hospital admission, before contact with other patients, and was designated as a community-acquired isolate.

In December 2022, two infants in the cardiac ward had a bloodstream infection with an ertapenem-resistant strain of *Kpn*, which alerted clinicians about a potential clonal outbreak. An outbreak investigation was initiated including retrospective analysis of faecal surveillance swabs for MDRO from all the infants and children who were inpatients in the cardiac and critical care wards. This investigation identified that between April and December 2022, a total of 11 infants and children (median age: 6 months, range: 0.5–28 months, including the two initially identified i.e. Patients 1 and 9) were colonised (n = 9), colonised and infected (n = 2) or infected (n = 1) with an apparently similar ertapenem resistant strain of *Kpn* belonging to ST45. An overview of the timeline of detections is shown in Figure 1. The outbreak occurred in the cardiac ward, but some of the affected patients had been transferred from the critical care ward (PICU) to the cardiac ward.

The isolates were designated as carbapenem-resistant Enterobacterales (CRE) based on resistance to ertapenem using E-test antimicrobial susceptibility testing (AST). Resistance to ciprofloxacin, tigecycline, ceftolozane/tazobactam and cefiderocol was also observed (Table). 

**Table:** Antimicrobial susceptibility profiles from routine testing performed following *Klebsiella pneumoniae* isolation in the hospital, United Kingdom, February–December 2022 (n = 12)

Patient	Sample ID	ERT	MER	CIP	GEN	AMI	TIG	CTV	CTX	FOX	CTT	FDC
Patient 1	MB1	1	≤ 0.25	R	S	S	R	S	R	R	R	S
Patient 2	MF1	1	0.25	R	S	S	R	S	R	R	R	S
Patient 3	MF2	1	1	R	S	S	ND	S	R	R	R	S
Patient 4-CA^a^	MF3-CA	1	0.25	R	S	S	S	S	R	R	R	S
Patient 5	MF4	1	0.25	R	S	S	R	S	R	R	R	R
Patient 6	MF5	1	0.25	R	S	S	R	S	R	R	R	R
Patient 7^b^	MF6	> 32	4	R	S	S	R	S	R	R	R	S
Patient 8	MF7	1	0.12	R	S	S	R	S	R	R	R	S
Patient 9	MB2	2	≤ 0.25	R	S	S	R	S	R	R	R	S
Patient 10	MF8	0.5	0.12	R	S	S	R	S	R	R	R	S
Patient 11^c^	MF9	1	0.12	R	S	S	R	S	R	R	R	S
Patient 12	MF10	1	0.12	R	S	S	S	S	R	R	R	S

AMI: amikacin; CA: community-acquired; CIP: ciprofloxacin; CTT: ceftolozane/tazobactam; CTV: ceftazidime/avibactam; CTX: cefotaxime; ERT: ertapenem, FDC: cefiderocol; FOX: cefoxitin; GEN: gentamicin; *Kpn: Klebsiella pneumoniae*; MB: microbiology sample from blood culture; MER: meropenem; MF: microbiology sample from faecal surveillance; ND: no data; R: resistant; S: susceptible; TIG: tigecycline.

^a^ The isolate from Patient 4 was positive for ertapenem-resistant *Kpn* at the time of hospital admission, before contact with other patients, and was designated as a community-acquired isolate.

^b^ The isolate from Patient 7 was subsequently found to be the co-isolation of *Kpn* and *Enterobacter hormaechei*.

^c^ The peritoneal fluid sample from Patient 11 contained both *Kpn* and *Enterococcus faecalis*. However, a pure *Kpn* culture could be isolated.

Antimicrobial susceptibility profiles from routine antimicrobial susceptibility testing (AST) performed following bacterial isolation from bloodstream cultures and faecal samples in the hospital are shown. ERT testing was performed using E-tests and MER testing was performed either using an E-test or the disc diffusion method. All other antimicrobials were tested using the disc diffusion method. Breakpoints for ERT in mm: S ≤ 0.5, R > 0.5; breakpoints for MER: S < 2, R > 8.

An additional patient had a positive faecal screening sample (Patient 4) for an ertapenem-resistant *Kpn* at the time of hospital admission, before contact with other patients. This was designated as a community-acquired isolate.

### Laboratory diagnostics, antimicrobial resistance phenotypes and clinical outcomes

The similar antibiogram profiles of the isolates and the proximity of the patients within the same hospital ward indicated potential nosocomial transmission. This was later confirmed by MLST performed by the UK Health Security Agency (UKHSA) at the national reference unit, which indicated all isolates to be ST45. 

Among the 12 affected patients, nine patients were colonised with CRE *Kpn* but did not exhibit signs of clinical illness, while three developed invasive infections. Two patients (Patient 1 and 9) developed bacteraemia with central line-associated bloodstream infections, one with previous faecal carriage (Patient 1) and one with negative faecal screening (Patient 9). One additional patient (Patient 11) was diagnosed with an intestinal perforation from necrotising enterocolitis leading to peritonitis with *Kpn* and *Enterococcus* isolated from the peritoneal fluid. Patient 11 had a previous positive faecal screening result. Patients with clinical illness responded to a combination of gentamicin, teicoplanin and cefiderocol treatment (Patient 1), gentamicin, teicoplanin and ceftazidime-avibactam (Patient 9) and meropenem plus surgery for the patient with peritonitis (Patient 11).

Hospital AST using the disc diffusion method identified isolates from two patients (Patient 5 and Patient 6) as resistant to cefiderocol, according to EUCAST clinical breakpoints (zone diameter < 23 mm). However, repeat testing using the broth microdilution method subsequently determined all isolates to be clinically susceptible (minimum inhibitory concentration (MIC) ≤ 2 μg/μL). The isolate from Patient 5 had an MIC value of 1 μg/μL which was a minimum of four times greater than the other isolates, which had MIC values or either 0.25 μg/μL or < 0.125 μg/μL.

The isolate from Patient 7 demonstrated a notably higher MIC for ertapenem (32 μg/μL) compared with other isolates (0.5–2 μg/μL). Initial genome sequencing quality control revealed the co-isolation of *Kpn* and *Enterobacter hormachei*. Due to repeated unsuccessful attempts to culture *Kpn* in isolation, this sample was excluded from further analysis. A similar co-isolation occurred in the peritoneal fluid sample from Patient 11 containing both *Kpn* and *Enterococcus faecalis*.

### Outbreak control measures

A number of outbreak control measures were initiated in the cardiac ward during December 2022 following recognition of the two children with bacteraemia that triggered an investigation by the infection prevention and control team. Following this, a retrospective review of faecal surveillance results over the previous year was performed, in combination with a search in the laboratory electronic information system for ertapenem- or meropenem-resistant *Kpn* isolates from patients from these wards. Screening of each patient for faecal carriage of antimicrobial resistant Gram-negative bacteria at admission and then weekly during hospitalisation was routine policy on the critical care ward but not the cardiac ward where it was introduced. Most children on the  wards were already in single occupancy side rooms, since the ward policy mandated that those colonised with carbapenem-resistant Gram-negative bacteria should be isolated in those rooms with contact precautions. 

These policies were reinforced, with education by the infection prevention and control team of staff, patients and their visitors about the detection of the resistant *Kpn* and how to prevent its transmission. A programme of enhanced personnel hand hygiene, personnel contact precautions, and increased environmental cleaning and decontamination was instituted. 

All medical equipment that was potentially used between patients and that could have contributed to nosocomial transmission was reviewed and sent to the decontamination department for further cleaning but was not tested for contamination with *Kpn*. Thermometers and scales used to weigh diapers were identified as potential contributors to nosocomial transmission events, as these pieces of equipment were found visibly not completely clean when dissembled and disinfected by the hospital decontamination team. The cardiac and critical care wards are not water-free. There is regular monitoring for *Pseudomonas aeruginosa* and *Legionella pneumophila* in the critical care unit sinks and checks of water temperature throughout the hospital water system per Health Technical Memorandum (HTM) 04-01. Although it is possible that colonisation of water sinks was potentially a source of transmission, the outbreak stopped despite no specific interventions in this area. 

New thermometers and a weighing scale for each patient room were purchased. The infection prevention and control team conducted daily rounds in the wards to ensure those under contact precautions were isolated and to monitor staff adherence to good practice. Following the institution of these measures during December, no additional patients on the ward became colonised or infected with the outbreak strain. The outbreak was detected for a total of 46 weeks.

### Genomic characterisation of antimicrobial resistance

Initial analysis of short-read sequencing data revealed that all isolates belonged to ST45 and were predicted capsule (K)-locus 62 and LPS O-antigen locus 01/02v1; all isolates were also predicted to contain an IncFIB(K) plasmid. The genomes contained several antibiotic ARGs, including *aph(3”)-lb*, *aph(6)-ld*, *bla*_DHA-1_ and the regulator *ampR*, *bla*_SHV-98_, *bla*_TEM-1_, *dfrA14*, *fosA*, *mphA*, *oqxA*, *oqxB*, *qacE*, *qnrB4*, *sul1* and *sul2*. This pattern was consistent across all isolates, except for isolate MF1 where *aph(3”)-lb*, *aph(6)-ld*, and *sul2* were not detected. Several non-synonymous chromosomal mutations were identified in the porin genes *ompK36* and *ompK37*, all of which were shared across the isolates. In *ompK36*, ten mutations were detected, including p.A217S, which is associated with carbapenem resistance; the remaining nine have been linked to cephalosporin resistance. In *ompK37*, three mutations (p.I70M, p.I128M, and p.N230G) were identified, all are associated with carbapenem resistance. No mutations were detected in *ompK35*. Additionally, seven non-synonymous chromosomal mutations were found in *acrR*, which encodes a multidrug efflux regulator; these mutations were present in all isolates and induce fluoroquinolone resistance. Isolates MF2 and MF1 also contained the S83F mutation in GyrA. We did not identify any specific genomic determinants that explained the observed differences in resistance profiles between isolates (Figure 1B) to ertapenem, cefiderocol and tigecycline. None of the strains were predicted to contain the *rmpA gene*, which can induce a hypermucoid phenotype. However, all isolates were positive for the yersiniabactin gene cluster, encoding for a siderophore-dependent iron uptake system, in the configuration ICEKP4/*ybt*10.

**Figure 2:**
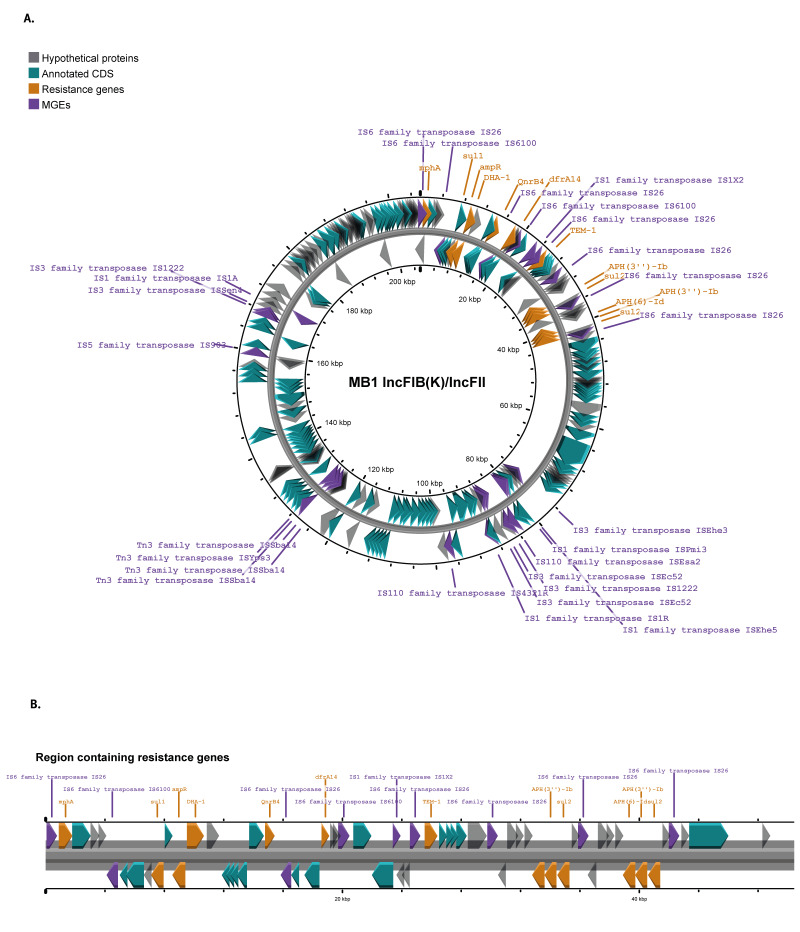
Detailed genetic background of the plasmid from the isolate MB1 driving the outbreak of *Klebsiella pneumoniae* in the hospital, United Kingdom, April 2022–February 2023

CDS: annotated coding sequences; MGEs: mobile genetic elements.

To resolve the structure and genomic location of the resistance determinants, three isolates, MB1 (from Patient 1), MF3-CA (community-acquired isolate from Patient 4), and MF9 (from Patient 11, the final patient admitted to the ward who later became colonised), were sequenced using both long- and short-read technologies. Assembly of isolate MB1 resulted in a circular chromosome of 5,252,750 bp, a circular IncFIB(K)/IncFII plasmid of 205,622 bp (Figure 2A), and an additional smaller plasmid of 149,366 bp classified as IncFIA(pBK30683)/IncFII(K). Additionally, two small linear contigs measuring 30,573 bp and 15,254 bp were assembled, neither of which contained any ARGs.

A. Schematic of the conjugative IncFIB(K) plasmid found in all outbreak *Klebsiella pneumoniae* isolates, with genes coding for antimicrobial resistance-inducing proteins and mobile genetic elements (MGEs) annotated.

B. The location of the resistance cassette containing the beta-lactamase genes *bla*_DHA-1_ and *bla*_TEM-1_ within the plasmid is shown along with all additional annotated coding sequences (CDS) within the resistance cassette.

The larger IncFIB(K)/IncFII plasmid (Figure 2A) harboured multiple ARGs, including the beta-lactamase genes *bla*_DHA-1_ and *bla*_TEM-1_. These genes were situated within a 42,625 bp region encoding resistance genes (Figure 2B), flanked by IS*26* elements. No additional resistance genes were identified outside this region on the plasmid; however, genes linked to metal resistance and other mobile genetic elements (MGEs) were present outside the region on the plasmid. The ARGs identified on the chromosome included *bla*_SHV-98_, *fosA*, *oqxA*, and *oqxB*. Apart from *aph(3”)-Ib*, *aph (6)-Id*, and *sul2*, which were duplicated on the plasmid, there was no evidence of amplification of other ARGs.

**Figure 3:**
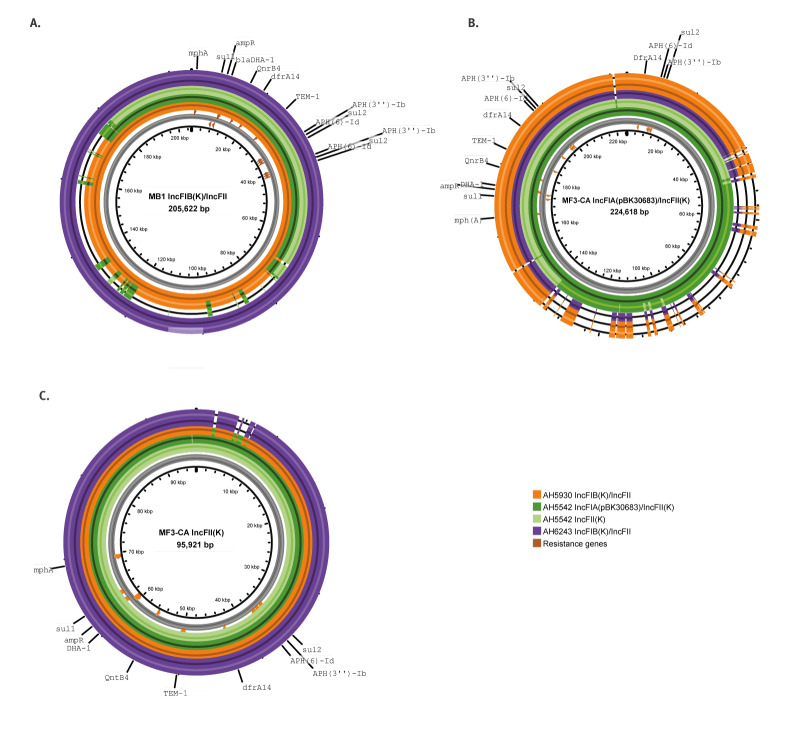
Comparative BLAST analysis of IncF plasmids showing the location of antimicrobial resistance genes from *Klebsiella pneumoniae* isolates driving the outbreak in the hospital, United Kingdom, April 2022–February 2023

Comparisons of the genomes constructed using long-read sequencing revealed notable variations in genome size, particularly in the community-acquired isolate MF3-CA. The total genome sizes for isolates MB1 and MF9 were 5,653,565 bp and 5,681,092 bp, respectively, while MF3-CA had a larger genome at 5,738,521 bp. A detailed comparison of the plasmids containing resistance genes (Figure 3) highlighted a high degree of similarity between the plasmids of MB1 and MF9, which were 205,622 bp and 205,641 bp, respectively, and both typed as IncFIB(K)/IncFII.

Concentric rings represent the IncFIB(K)/IncFII plasmid from isolate MB1 (from the first known colonised patient, purple), the larger IncFIA(pBK30683) plasmid from isolate MF3-CA (the community-acquired isolate, dark green), the smaller IncFII(K) plasmid from isolate MF3-CA (light green), and the IncFIB(K)/IncFII plasmid from MF9 (from the final patient admitted to the ward who later became colonised, orange). Panel A shows the three other plasmids compared with the IncFIB(K)/IncFII from isolate MB1. Panel B shows all other plasmids compared with the IncFIA(pBK30683) plasmid from isolate MF3-CA. Panel C shows all other plasmids compared with the IncFII(K) plasmid from isolate MF3-CA. The ARGs are annotated based on their position in the reference sequence, to which all other plasmid sequences were aligned.

In contrast, two plasmids harbouring ARGs were identified in MF3-CA. The larger plasmid, 224,681 bp in size, was typed as IncFIA(pBK30683)/IncFII(K), while the smaller plasmid, typed as IncFII(K), measured 95,921 bp. Both plasmids in MF3-CA contained all the same ARGs as those in MB1 and MF9, though the arrangement of genes varied slightly (Figures 3B and 3C). Notably, in the smaller IncFII(K) plasmid, *aph(3”)-Ib, aph(6)-Id*, and *sul2* were not duplicated. Despite its reduced size, this smaller plasmid shared a high degree of similarity with the larger plasmid from MF3-CA (Figure 3C). It also resembled the plasmids from MB1 and MF9, except for a small region between 2 kb and 8 kb where sequence homology was not conserved. The larger plasmid in MF3-CA contained unique regions that were absent in the plasmids from MB1 and MF9, while also lacking specific regions present in those isolates. Despite these structural differences, sequence identity was notably conserved across all plasmids in regions containing the ARGs.

**Figure 4:**
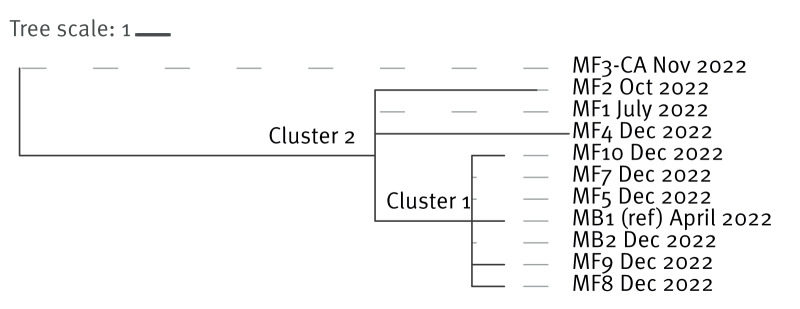
Detailed phylogenetic tree of *Klebsiella pneumoniae* isolates from the outbreak in the hospital, United Kingdom, April 2022–February 2023 (n = 11)

CA: community-acquired isolate; ref: reference; SNV: single nucleotide variant.

To identify sub-lineages within the clonal *Kpn* outbreak collection, we employed a root-to-tip directional approach based on SNV distances from the ancestral node when constructing a phylogenetic tree (Figure 4) [39]. Isolate MB1, obtained from the first known colonised patient formed a distinct cluster (Cluster 1) with the majority of isolates obtained from patients colonised and/or infected in December 2022 (MF10, MF7, MF5, MB2, MF9 and MF8), highlighting a high level of clonality and a close genomic relationship among these isolates. Cluster 2 comprises a distinct subset of strains that are more closely related to each other than to the first collected isolate or the isolates in Cluster 1. This cluster includes isolates MF1, MF2 and MF4, which were obtained from patients colonised in July 2022, October 2022 and December 2022, respectively. The patients from whom isolates MF1 and MF2 were collected were the next two admissions to the ward, in March 2022 and August 2022, respectively, following the first patient (from whom isolate MB1 was obtained). Despite their temporal proximity, these isolates do not cluster with MB1. The community-acquired isolate (MF3-CA) exhibited the greatest divergence from all other isolates, and based on our classification, it represents a separate major lineage.

rPinecone tree constructed using a root-to-tip directional approach, according to SNV distance from the ancestral node. Isolate MB1 was used as a reference. New minor lineages were defined by 5 SNVs, and new major lineages were defined by 10 SNVs, when compared with the reference.

## Discussion

Our investigation offers crucial genomic insights into a prolonged clonal outbreak of *Kpn* in a paediatric cardiac unit, where resistance to the carbapenem antibiotic ertapenem and the beta-lactam/beta-lactamase inhibitor drug ceftolozane/tazobactam was likely driven by beta-lactamases SHV-98, TEM-1 and DHA-1 in combination with potential alterations in porin structure given multiple non-synonymous changes in the sequences of *ompK36* and *ompK37.* Although, to our knowledge, there are no studies that definitively demonstrate the impact of the mutations identified on porin tertiary structure, the *ompK36* p.A217S mutation and the *ompK37* mutations p.I70M, p.I128M and p.N230G observed in our study have also been reported in ertapenem-resistant *Kpn* isolates lacking carbapenemase production [41]. The *acrR* mutations identified in our study have previously been reported in fluoroquinolone-resistant *Kpn* isolates, and their role in conferring resistance has been experimentally validated [42]. Genomic analyses confirmed that all hospital-derived isolates were part of a clonal outbreak, belonging to ST45, and were of capsule type K62 and LPS O-antigen type O2a. *Klebsiella pneumoniae* ST45 is a globally distributed, multidrug-resistant ST associated with healthcare-acquired transmission clusters [10] and implicated in hospital outbreaks resulting in invasive infections, especially in vulnerable populations, along with concurrent environmental contamination [43]. ST45 has been found to harbour ESBLs and carbapenemases, and resistance genes to other drug classes such as fluoroquinolones, tetracyclines and colistin [12].

The beta-lactamase ARGs associated with ceftolozane/tazobactam and ertapenem resistance were both chromosomally encoded (*bla*_SHV-98_) and plasmid-encoded (*bla*_DHA-1_ and *bla*_TEM-1_) and are capable of hydrolysing beta-lactam antibiotics to varying degrees. Notably, because of cumulative mutations, SHV-98 can exhibit an extended-spectrum beta-lactamase (ESBL) phenotype and DHA-1, a plasmid-mediated AmpC beta-lactamase, is linked to resistance against piperacillin/tazobactam [44] and carbapenems, especially when co-expressed with other beta-lactamases [45] and in isolates with reduced porin expression [46], as observed in our study. The plasmid-mediated expression of *bla*_DHA-1_ is regulated by the upstream *ampR* gene [47]. In our isolates, this configuration potentially leads to an expanded resistance spectrum, including resistance to ertapenem.

Phenotypic tigecycline resistance was detected in the isolates. However, none of the known mechanisms could be identified in this study, but tigecycline resistance has recently been observed to be linked to changes in porins, highlighting the complexity of detecting these as they might be driven by changes in transcriptional or translational regulation and not apparent in the gene sequences themselves [48,49]. Tigecycline is typically indicated for complicated skin and soft-tissue infections, and complicated intra-abdominal infections, where other antimicrobials are not suitable [50]. Although tigecycline is contraindicated in young children and infants, its off-label use has been documented in paediatric patients with infections caused by extensively drug-resistant Gram-negative bacteria [51], and it has previously been effective against ESBL-producing and AmpC-producing Enterobacterales [52]*.* Reporting these emerging resistance mechanisms and their association with ESBL-producing or carbapenem-resistant Enterobacterales is crucial for early identification and management of tigecycline resistance.

Many of the ARGs identified were plasmid-mediated and located in regions flanked by IS*26* elements, highlighting the potential for horizontal gene transfer and the further spread of resistance among Enterobacterales. The ARG-containing IncFIB(K)/IncFII plasmids in isolates MB1 and MF9 were highly similar. However, in the community-acquired MF3-CA isolate, ARGs were located on both an enlarged version of the IncFIB(K)/IncFII plasmid (typed as IncFIA(pBK30683)/IncFII(K)) and on a smaller IncFII(K) plasmid. Despite the size differences, the resistance region remained intact on both plasmids. The presence of IS*26* elements flanking the resistance region likely facilitates its mobilisation and could have facilitated the transfer of the region between plasmids within the same isolate. This mechanism has been previously described for plasmid reorganisation in clinical isolates with multiple ARGs [53]. Furthermore, interactions between conjugative plasmids facilitated by IS elements have been identified as a general mechanism by which the transfer of ARGs is mediated [54].

Additional genomic differences were identified in the community-acquired MF3-CA isolate when compared with the other hospital-associated isolates. Comparative analysis of core genomes revealed that the community-acquired isolate (MF3-CA) was more distantly related to the hospital-associated strains, with a 10 SNP distance to the closest hospital-acquired isolate (MF2), suggesting the *Kpn* strain is also circulating within the community.

A limitation of our study is that resistance mechanisms were inferred from genomic data with only AST used as phenotypic validation. This means we cannot definitively confirm that the observed resistance to ertapenem and ceftolozane/tazobactam was solely due to DHA-1, TEM-1 and SHV-98 production, nor can we determine if the beta-lactamases acted synergistically to induce resistance. Matching unusual genotypes to phenotypic resistance is particularly important when considering the design of molecular diagnostics. While molecular CRE surveillance assays commonly screen for carbapenemase genes, our study emphasises the importance of identifying unusual genotypes that induce carbapenem resistance, which might otherwise be missed by current diagnostics. To improve detection of such strains in the future, targeted genomic screening or sequencing of isolates with atypical resistance phenotypes—or phenotypes of particular public health concern (such as CRE) may be beneficial. This approach can help identify non-canonical resistance mechanisms, including those where synergism results in resistance, that are often missed by standard molecular assays. While methods such as WGS are not currently suited for routine diagnostics due to high turnaround time and costs compared to routine molecular typing, their ability to provide high-resolution SNP typing and structural context for mobile resistance elements makes them a valuable tool for surveillance, and their increasing accessibility allows for rapid deployment when outbreaks are suspected.

A further limitation is that this is a retrospective study. Patients changed beds/rooms between the cardiac ward and the critical care ward through their hospital course, and there is no electronic tracking system for movement of staff or relatives. We were furthermore not able to confirm if the medical equipment, ward surfaces or sinks were contaminated with the outbreak organism or determine whether *Kpn* remained in the environment, as the outbreak was considered stopped when no additional faecal surveillance samples were positive for ertapenem-resistant *Kpn*.

The outbreak’s duration and the identification of both community-acquired and healthcare-associated isolates highlight the challenges of eradicating *Kpn* strains from clinical settings. An investigation into the outbreak suggested that shared equipment such as thermometers and weighing scales may have facilitated transmission between patients and the enhancement implementation of standard infection control practices and the introduction of patient-dedicated equipment subsequently ended the outbreak. This mirrors findings from a previous study describing an outbreak of *Kpn* ST45 in neonatal intensive care units, which was also associated with environmental contamination [43]. The observed bloodstream infections are hypothesised to derive from initial colonisation as detected in Patient 1, whereas Patient 9 lacked prior colonisation and most likely acquired the infection via an environmental source. In Patient 11, the most likely route was gastrointestinal colonisation at the time of perforation of the peritoneum caused by necrotising enterocolitis. 

## Conclusions

Identifying phenotypic patterns, such as AST profiles, along with patient location data, is crucial for outbreak detection and management. The mosaic nature of mobile elements carrying resistance determinants, combined with synergistic and antagonistic effects of these on each other, further complicates genome-based predictions and highlights the relevance of regular phenotyping to characterise novel combinations leading to unexpected phenotypes. Combining genomic investigations with phenotypic data provides a comprehensive approach to understanding the spread of resistant bacteria and mitigating further dissemination, especially in clinical settings. This combinatorial approach is essential for strengthening healthcare systems and managing risks posed by multidrug-resistant organisms, particularly in vulnerable patient populations.

## Data Availability

Illumina and ONT reads can be found at the Sequence Read Archive bioproject ID PRJNA1211177. A detailed list of accession numbers is available in Supplementary Table S1.

## Supplementary Data

48000 Supplement
