# Supplementary material for: A 46-week outbreak of ertapenem-resistant, non-carbapenemase encoding Klebsiella pneumoniae ST45 in a paediatric cardiac unit involving shared equipment, United Kingdom, April 2022 to February 2023
Source: Euro Surveill. 2025 Oct 30;30(43):2500133. doi: 10.2807/1560-7917.ES.2025.30.43.2500133 (PMC12579315; doi:10.2807/1560-7917.ES.2025.30.43.2500133)
Supplement: Supplement [file 25-00133_HEINZ_Supplementary_tableS1.pdf]

This supplementary material is hosted by Eurosurveillance as supporting information alongside the article **[A 46-week outbreak of ertapenem-resistant, non-carbapenemase encoding *Klebsiella pneumoniae* ST45 in a paediatric cardiac unit involving shared equipment, United Kingdom, April 2022 to February 2023]** on behalf of the authors who remain responsible for the accuracy and appropriateness of the content. The same standards for ethics, copyright, attributions and permissions as for the article apply. Eurosurveillance is not responsible for the maintenance of any links or email addresses provided therein.

ID	short read accession	long read accession	sample ID
MB1	SRR32026274	SRR32026277	SAMN46269047
MF1	SRR32101185		SAMN46389068
MF2	SRR32101184		SAMN46389069
MF3-CA	SRR32026273	SRR32026276	SAMN46269048
MF4	SRR32101183		SAMN46389070
MF5	SRR32101182		SAMN46389071
MF6	SRR32101181		SAMN46389072
MF7	SRR32101180		SAMN46389073
MB2	SRR32101179		SAMN46389074
MF8	SRR32101178		SAMN46389075
MF9	SRR32026272	SRR32026275	SAMN46269049
MF10	SRR32101177		SAMN46389076
